# Hydroxysafflor Yellow A for Diabetic Retinopathy: A Critical Review of Retinal Neurovascular Mechanisms and Systemic-to-Ocular Pharmacokinetic Barriers

**DOI:** 10.3390/antiox15070865

**Published:** 2026-07-10

**Authors:** Jiaqi Liu, Wenjing Liu, Lu Li, Qianqian Zhang, Jun Zhang, Wenjie Yan

**Affiliations:** College of Biochemical Engineering, Beijing Union University, Beijing 100023, China; liujiaqi0711@foxmail.com (J.L.); 18438191289@163.com (W.L.); lilu_academic@163.com (L.L.); 15942376311@163.com (Q.Z.); 13883341384@163.com (J.Z.)

**Keywords:** hydroxysafflor yellow A, oxidative stress, retinal neurovascular unit, diabetic retinopathy, blood–retinal barrier, ocular pharmacokinetics, drug delivery

## Abstract

Oxidative stress contributes to retinal neurovascular injury through inflammation, mitochondrial dysfunction, blood–retinal barrier (BRB) disruption, microcirculatory impairment, and regulated cell death. Hydroxysafflor yellow A (HSYA), a water-soluble quinochalcone C-glycoside derived from safflower (*Carthamus tinctorius* L.), modulates oxidative and inflammatory signaling, apoptosis, mitochondrial injury, endothelial barrier dysfunction, and neurovascular damage in experimental ischemic, inflammatory, and metabolic disorders. This review critically evaluates the direct ocular evidence for HSYA in diabetic retinopathy and examines the systemic-to-ocular pharmacokinetic and delivery barriers that constrain its ophthalmic translation. Current ocular evidence is limited and concentrated mainly in DR models, in which HSYA attenuates oxidative stress, inflammation, BRB disruption, and apoptosis, potentially through Nrf2/HO-1 signaling. Evidence in retinal photic injury is limited, whereas the proposed relevance of HSYA to retinal ischemia–reperfusion injury, glaucoma, and AMD remains largely hypothesis-generating. The principal translational challenge is whether HSYA can achieve pharmacologically relevant exposure in ocular target tissues. Future studies should integrate dose, plasma and ocular exposure, target engagement, retinal structure, local safety, and visual function in disease-specific models. Accordingly, evidence from non-DR models is discussed primarily to define mechanistic hypotheses and experimental priorities rather than to establish ophthalmic efficacy.

## 1. Introduction

Population ageing has increased the burden of age-related ocular diseases. Ageing-related changes in ocular tissues are accompanied by oxidative stress, impaired proteostasis, mitochondrial dysfunction, and chronic inflammation, contributing to AMD, cataract, glaucoma, DR, dry eye disease, and RP [1,2,3,4]. Targeting oxidative damage and mitochondrial dysfunction has therefore become an important strategy for the prevention and treatment of ocular diseases [5].

Oxidative stress is a shared pathological feature underlying the development and progression of numerous ocular diseases. The retina is particularly susceptible to reactive oxygen species (ROS)-mediated injury because of its high oxygen consumption, continuous exposure to light, abundance of polyunsaturated fatty acids, and intense metabolic activity [6,7]. When ROS production exceeds the capacity of endogenous antioxidant defenses, it can trigger lipid peroxidation, protein oxidation, DNA damage, mitochondrial dysfunction, inflammatory responses, and cell death [8,9]. Varying degrees of redox imbalance have been documented in diabetic retinopathy (DR), age-related macular degeneration (AMD), glaucoma, retinal ischemia–reperfusion injury, and light-induced retinal damage. Consequently, targeting oxidative stress and its downstream inflammatory, apoptotic, and mitochondria-associated pathways has become a central focus in the development of interventions for oxidative stress-related ocular diseases [10].

**Figure 1:**
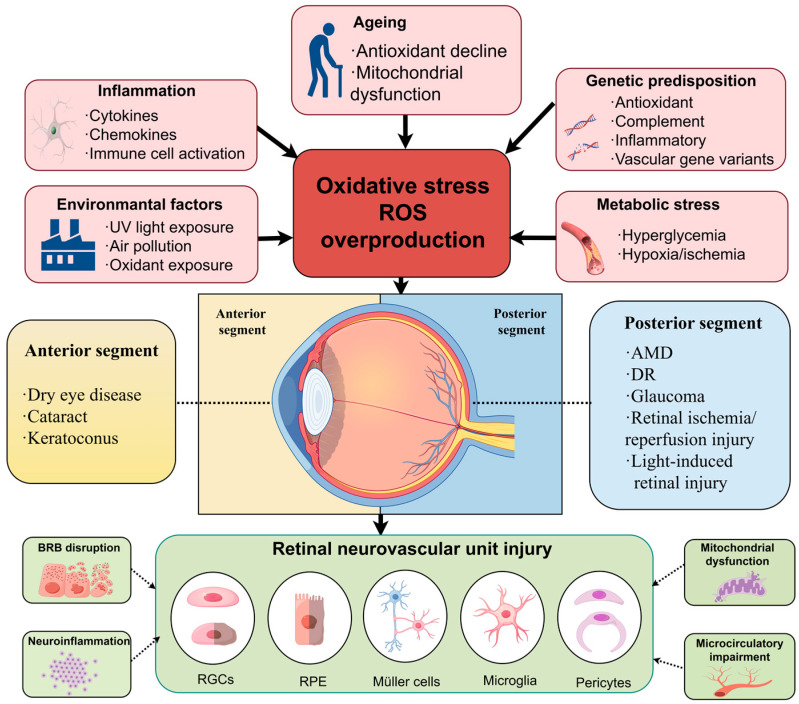
Oxidative stress-driven ocular injury and its convergence on retinal neurovascular unit dysfunction. Aging, genetic predisposition, inflammation, environmental exposure, and metabolic stress promote reactive oxygen species (ROS) overproduction, contributing to anterior and posterior segment diseases. In the posterior segment, oxidative stress can disrupt the blood-retinal barrier (BRB), induce neuroinflammation, impair mitochondrial function and microcirculation, and ultimately damage retinal ganglion cells (RGCs), retinal pigment epithelial (RPE) cells, Müller cells, microglia, endothelial cells, and pericytes. (https://www.figdraw.com/static/index.html#/, accessed on 30 June 2026).

In recent years, the conceptual framework for ocular disease pathogenesis has shifted from isolated cellular injury toward dysfunction of the neurovascular unit as an integrated system [11]. In the retina, retinal ganglion cells (RGCs), retinal pigment epithelial (RPE) cells, Müller glia, microglia, vascular endothelial cells, and pericytes interact to maintain metabolic homeostasis, barrier integrity, and microcirculatory function [12,13,14]. Oxidative stress not only directly damages retinal neurons and RPE cells but also activates inflammatory responses mediated by microglia and Müller cells, disrupts the blood–retinal barrier (BRB), and promotes endothelial dysfunction and microvascular abnormalities [15]. These processes establish a self-reinforcing cycle in which neurodegeneration and vascular pathology progressively exacerbate one another. Natural bioactive compounds capable of concurrently modulating oxidative stress, inflammation, mitochondrial injury, and neurovascular unit homeostasis may therefore represent promising candidates for ophthalmic research. The oxidative stress-driven injury pathways and their convergence on retinal neurovascular unit dysfunction are summarized in Figure 1.

Against this background, naturally occurring antioxidant compounds derived from medicinal plants have attracted increasing attention. Hydroxysafflor yellow A (HSYA) is one of the principal water-soluble bioactive constituents of *Carthamus tinctorius* L. and belongs to the quinochalcone C-glycoside class. Previous studies have attributed a broad range of pharmacological activities to HSYA, including antioxidant, anti-inflammatory, anti-apoptotic, microcirculation-improving, mitochondrial-protective, and neurovascular-protective effects. Accordingly, HSYA has been extensively investigated in experimental models of cerebral ischemia, myocardial ischemia, inflammatory injury, and metabolic disorders [16,17]. These reported activities overlap substantially with key pathological processes implicated in oxidative stress-related ocular diseases, including ROS accumulation, amplification of inflammatory signaling, apoptosis, endothelial injury, disruption of the blood–retinal barrier, and microcirculatory dysfunction. This mechanistic convergence provides a rationale for further evaluating HSYA as a natural antioxidant candidate in ophthalmic research.

Existing reviews of HSYA have focused primarily on its chemical characteristics, systemic pharmacological activities, cardiovascular and cerebrovascular protection, antioxidant and anti-inflammatory mechanisms, or general pharmacokinetic and drug-delivery properties. By contrast, few studies have integrated the available evidence from the perspectives of ocular oxidative stress, retinal neurovascular unit injury, and drug exposure in ocular tissues. Importantly, direct ophthalmic evidence for HSYA is currently concentrated mainly in models of diabetic retinopathy, whereas its proposed effects in retinal photic injury, retinal ischemia–reperfusion injury, glaucoma, and age-related macular degeneration remain largely extrapolated from non-ocular models or inferred from shared mechanisms. The ophthalmic translational potential of HSYA, therefore, cannot be judged solely on the basis of its antioxidant activity or systemic pharmacology. Critical questions include whether HSYA reaches target tissues such as the retina and retinal pigment epithelium, achieves sufficient and sustained ocular exposure, engages key pathological processes within the retinal neurovascular unit, and can be delivered through routes that provide acceptable local safety. Accordingly, this review is reframed primarily around diabetic retinopathy, for which direct ocular evidence is currently available, and around the systemic-to-ocular pharmacokinetic barriers that limit ophthalmic translation. Evidence from retinal photic injury, retinal ischemia–reperfusion injury, glaucoma, and AMD is considered mainly as hypothesis-generating context and not as proof of disease-specific efficacy. This framework is intended to provide a more clinically and pharmacologically grounded basis for future ophthalmic development of HSYA.

## 2. Search Strategy and Evidence Classification

This review was designed as a structured narrative review. Literature was searched in PubMed, Web of Science, Scopus, Google Scholar, and CNKI, with the last search performed on 20 May 2026. Search terms included combinations of “hydroxysafflor yellow A”, “HSYA”, “safflower”, “Carthamus tinctorius”, “oxidative stress”, “antioxidant”, “Nrf2”, “retina”, “retinal pigment epithelium”, “retinal ganglion cell”, “diabetic retinopathy”, “age-related macular degeneration”, “glaucoma”, “retinal light injury”, “ischemia-reperfusion injury”, “blood-retinal barrier”, “pharmacokinetics”, “ocular delivery”, and “drug delivery”.

Studies were eligible when they addressed the chemistry, redox pharmacology, antioxidant mechanisms, safety, pharmacokinetics, delivery, ocular effects, or ocular-relevant biological effects of HSYA. Primary studies were prioritized over reviews when evaluating pharmacological mechanisms and disease-related evidence. Reviews were used mainly to contextualize disease mechanisms, oxidative stress biology, and drug delivery barriers.

The included evidence was organized according to its relevance to ophthalmic translation. Direct ocular evidence was defined as studies using ocular cells, retinal tissues, or ophthalmic animal models. Ocular-relevant indirect evidence was defined as studies using non-retinal neurovascular, endothelial, inflammatory, ischemic, mitochondrial, barrier-related, or cell-death-related models with mechanistic relevance to retinal injury. Findings from non-ocular models were not interpreted as evidence of ocular efficacy but were used to identify mechanistic hypotheses and validation priorities for future retinal studies.

Because this article is a structured narrative review rather than a systematic review or meta-analysis, formal risk-of-bias assessment and quantitative evidence synthesis were not performed. Accordingly, the conclusions are framed as mechanistic rationale, evidence interpretation, and translational research priorities rather than clinical recommendations.

## 3. Chemical, Pharmacological, and Safety Basis of HSYA

### 3.1. Major Active Constituents in Safflower

Safflower has a chemically diverse phytochemical profile. More than 300 compounds have been isolated or identified from different parts of the plant, predominantly flavonoids, organic acids, and alkaloids, while safflower seeds are also rich in fixed oils [18]. Among these constituents, flavonoids are the most extensively characterized class and are considered major contributors to the antioxidant [19], anti-inflammatory [20], vasoprotective [21], and neuroprotective activities of safflower [20]. Structurally, safflower flavonoids can be classified into several groups, including quinochalcones, flavonols, flavones, and flavanones [18]. Its physicochemical, pharmacokinetic, and safety profiles, which are particularly relevant to ophthalmic translation, are summarized in Table 1.

Safflower yellow pigments (SYs) are the most representative water-soluble pigments in safflower and consist primarily of quinochalcone C-glycosides. This group includes safflor yellow A (SYA), safflor yellow B (SYB), hydroxysafflor yellow A (HSYA), hydroxysafflor yellow B (HSYB), hydroxysafflor yellow C (HSYC), and anhydrosafflor yellow B (AHSYB) [22]. Among them, HSYA is the most extensively investigated individual constituent. Its well-defined chemical structure and high water solubility have established it as a major marker compound in the quality control and pharmacological evaluation of safflower. Fractionation studies have shown that SY preparations contain SYB, HSYA, HSYB, and other related water-soluble constituents, with HSYA regarded as a principal water-soluble quinochalcone C-glycoside pigment and an important quality marker of safflower [23].

In addition to safflower yellow pigments, safflower contains numerous flavonols, flavones, and their glycosides, particularly derivatives of quercetin, kaempferol, and apigenin [22,24]. These compounds commonly possess multiple phenolic hydroxyl groups and may contribute to the overall antioxidant capacity of safflower by scavenging free radicals, maintaining redox homeostasis, and modulating oxidative stress-responsive signaling pathways. Other constituents, including organic acids, alkaloids, polyacetylenes, and spermidine derivatives, may also act cooperatively in mediating the anti-inflammatory, antioxidant, and vascular-regulatory effects of safflower; however, these compound classes remain less extensively studied than flavonoids [18].

HSYA is derived predominantly from the flowers of safflower and represents one of its most characteristic water-soluble quinochalcone di-C-glycosides. Recent biosynthetic studies have demonstrated that HSYA accumulates preferentially in floral tissues and that its formation is closely associated with flavonoid and chalcone metabolism. Its biosynthesis involves a series of reactions, including the hydroxylation and isomerization of naringenin-related precursors, C-glycosylation, and oxidative rearrangement. Several key enzymes, including CtF6H, CtCHI1, CtCGT, and Ct2OGD1, have been shown to participate in this pathway [25,26]. These findings not only provide a biochemical explanation for the characteristic occurrence and tissue-specific accumulation of HSYA in safflower but also establish a foundation for its sustainable and efficient production through synthetic biology and metabolic engineering.

From the perspective of antioxidant activity and ophthalmic translation, safflower can therefore be regarded as a natural source of bioactive flavonoids, with HSYA serving as a representative constituent. Its defined botanical origin, distinctive chemical structure, and comparatively well-established pharmacological profile make HSYA an appropriate focal compound for examining the antioxidant properties of safflower, its potential effects on retinal neurovascular protection, and its prospects for ophthalmic development.

### 3.2. Chemical Structure and Physicochemical Properties of HSYA

HSYA has a molecular formula of C_27_H_32_O_16_ and a relative molecular mass of approximately 612.5 g/mol, and it is typically obtained as a yellow to orange-yellow powder. The presence of multiple phenolic hydroxyl and glycosyl groups confers high hydrophilicity and low lipophilicity. Consequently, HSYA is readily soluble in water but exhibits limited solubility in lipophilic organic solvents such as chloroform, benzene, and ethyl acetate [27]. Its high aqueous solubility facilitates water-based extraction and the development of injectable formulations. Conversely, its high polarity and low lipophilicity restrict passive membrane permeation, contributing to poor oral absorption and limited penetration across biological barriers [16,28].

The physicochemical stability of HSYA is also relatively limited. Its chalcone glycoside structure renders it sensitive to pH, temperature, and light, and degradation may occur under strongly acidic or alkaline conditions, elevated temperatures, or prolonged light exposure. Previous studies have indicated that light, heat, and alkaline environments can promote oxidative degradation, hydrolysis, or polymerization of HSYA [16]. These stability constraints complicate formulation development and storage, while its pronounced polarity further reduces transmembrane transport efficiency and may limit systemic and tissue exposure.

These physicochemical properties directly influence the extraction, formulation, and pharmacokinetic behavior of HSYA. Conventional aqueous extraction is suitable for water-soluble constituents such as HSYA, but it is often limited by relatively low extraction yields, high consumption of plant material, and the risk of thermal degradation during processing [29]. Consequently, increasing attention has been directed toward greener extraction technologies and strategies that improve the delivery and bioavailability of HSYA.

Natural deep eutectic solvents (NADESs), combined with ultrasound-assisted extraction, have been investigated as an alternative approach for recovering water-soluble bioactive constituents from safflower [30]. In a comparative screening of 22 NADES formulations, an L-proline–acetamide-based system markedly enhanced the extraction efficiencies of HSYA and anhydrosafflor yellow B (AHSYB). Animal experiments further showed that this formulation increased systemic exposure to both compounds following oral administration. These findings suggest that NADESs may serve not only as environmentally preferable extraction media but also as functional formulation vehicles capable of improving the in vivo availability of safflower yellow pigments [30].

From a pharmacological perspective, HSYA has become the most widely used representative constituent in studies investigating the antioxidant, anti-inflammatory, vasoprotective, and neuroprotective effects of safflower. Its phenolic hydroxyl groups and conjugated system may contribute to radical-scavenging activity and redox regulation, whereas its quinochalcone scaffold may underlie its capacity to interact with multiple pharmacological targets [29]. HSYA is also recognized as a principal bioactive constituent and an important quality-control marker of safflower. It has consequently attracted substantial research interest in cardiovascular and cerebrovascular disorders, inflammatory injury, neuroprotection, and cancer-related models [16].

HSYA is a well-characterized safflower constituent with documented antioxidant, anti-inflammatory, and neurovascular effects. Its ophthalmic development is limited by high polarity, low lipophilicity, poor stability, low oral bioavailability, and uncertain ocular exposure. Further progress will depend on formulations and delivery strategies that improve stability, barrier penetration, and drug levels in retinal target tissues.

### 3.3. Fundamental Pharmacological Activities of HSYA

Oxidative stress, inflammation, cell death, and microcirculatory dysfunction are shared pathological processes in several ocular diseases, including diabetic retinopathy, age-related macular degeneration, glaucoma, and cataract. Natural bioactive compounds with antioxidant, anti-inflammatory, anti-apoptotic, and vasoprotective properties may therefore provide potential therapeutic candidates for the intervention of oxidative stress-related ocular disorders [6].

As a representative water-soluble bioactive constituent of safflower, HSYA has demonstrated relatively consistent pharmacological activity across a range of experimental models of ischemic, inflammatory, and metabolic injury. Available evidence indicates that its protective effects involve attenuation of oxidative stress and inflammatory responses, suppression of apoptosis, modulation of autophagy, improvement of microcirculatory function, and preservation of vascular barrier integrity [17]. These findings suggest that the biological activity of HSYA cannot be attributed solely to direct radical scavenging. Rather, HSYA appears to modulate tissue injury through coordinated actions on multiple molecular targets and signaling pathways.

Direct ophthalmic evidence for HSYA is currently derived primarily from experimental models of diabetic retinopathy. In diabetic rats, HSYA has been reported to ameliorate retinal histopathological injury and blood–retinal barrier disruption, accompanied by improvements in markers of oxidative stress, inflammation, and apoptosis [31]. These findings suggest that its retinal protective effects may involve enhancement of Nrf2/HO-1-mediated antioxidant defense. This study therefore provides preliminary direct evidence supporting further investigation of HSYA in oxidative stress-related retinal diseases.

Beyond ocular models, studies of cerebral ischemia–reperfusion injury, intracerebral hemorrhage, myocardial ischemia–reperfusion injury, and vascular endothelial damage provide indirect support for the neurovascular-protective properties of HSYA [20]. These studies suggest that HSYA can modulate inflammatory signaling, microglial activation, mitochondrial function, vascular barrier integrity, and microcirculatory homeostasis [17,20,32]. Given that the retina and central nervous system share several biological features, including high oxygen demand, a neurovascular unit organization, and specialized barrier systems, findings from non-ocular models may offer mechanistic guidance for the ophthalmic investigation of HSYA. However, such evidence should not be interpreted as direct proof of therapeutic efficacy in ocular tissues or retinal disease [33].

Overall, the fundamental pharmacological profile of HSYA can be characterized by a multilayered protective network encompassing antioxidant, anti-inflammatory, anti-apoptotic, microcirculation-enhancing, and neurovascular-protective actions. These properties provide a plausible biological rationale, together with preliminary experimental support, for investigating HSYA in oxidative stress-related ocular diseases. Nevertheless, direct evidence remains insufficient in glaucoma, age-related macular degeneration, retinal ischemia–reperfusion injury, and other retinal disorders. Its ophthalmic potential therefore requires further critical evaluation at both the mechanistic level and within disease-specific experimental models, as discussed in the following sections.

### 3.4. Safety and Toxicity of HSYA

Available evidence generally indicates that HSYA is well tolerated and has a favorable safety profile within certain dose ranges. Nevertheless, its safety should be assessed in relation to the route of administration, dose, treatment duration, coagulation status, renal clearance, and potential drug–drug interactions. Given its reported anticoagulant, anti-inflammatory, and vascular-regulatory activities, HSYA should not be presumed to be a low-risk compound solely because it is derived from safflower.

Human studies provide important evidence regarding the safety profile of HSYA. A phase I clinical pharmacology study in healthy Chinese volunteers showed that intravenous HSYA was generally well tolerated. Most adverse events were mild, and no treatment discontinuations attributable to the study drug were reported. HSYA was eliminated relatively rapidly, with a terminal half-life of approximately 4.0–4.7 h [34]. However, coagulation-related changes, including prolongation of activated partial thromboplastin time (APTT), were also observed, indicating that coagulation parameters should be monitored during clinical administration.

Further evidence was obtained from a phase II multicenter, randomized, double-blind trial of HSYA injection in patients with acute ischemic stroke associated with blood stasis syndrome. Intravenous infusion of HSYA at 25, 50, or 70 mg/day for 14 consecutive days was generally safe and well tolerated, with no significant differences in the incidence of specified adverse events among the treatment groups [35]. These findings support a favorable short-term tolerability profile for intravenous HSYA within the evaluated dose range, although they do not establish its safety during prolonged treatment or through alternative routes of administration.

Although human studies indicate that short-term administration of HSYA is generally well tolerated, animal toxicology studies have further defined potential safety limits under conditions of prolonged or high-dose exposure. The principal safety concerns identified to date involve prolongation of coagulation time and renal injury. In a 90-day subchronic toxicity study, repeated intraperitoneal administration of HSYA at 60 or 180 mg/kg prolonged coagulation time in rats, although this effect was reversible after treatment discontinuation. Mild renal tubular injury was also observed in the 180 mg/kg group, indicating that coagulation status and renal function warrant particular attention during prolonged or high-dose administration [36].

With respect to reproductive and developmental toxicity, a recent study in Sprague–Dawley rats reported that HSYA administered at 25, 75, or 250 mg/kg/day did not significantly increase the incidence of malformations or developmental variations in the F1 generation. The no-observed-adverse-effect level was identified as 250 mg/kg/day, and HSYA was also reported to cross the placental and blood–milk barriers [37]. These findings underscore the need for careful risk assessment during pregnancy and lactation.

The pharmacokinetic properties of HSYA are also relevant to its safety profile. Owing to its high water solubility and low lipophilicity, HSYA has poor oral bioavailability and is cleared relatively rapidly after systemic administration [29,34]. HSYA-related metabolites have been identified in plasma, bile, urine, and feces after oral administration, but the quantitatively dominant excretion route has not been firmly established [38]. Rapid clearance may reduce the likelihood of long-term systemic accumulation; however, maintaining therapeutically relevant exposure may require adjustment of the dosing frequency, administered dose, or formulation. Renal burden and the potential for systemic accumulation should therefore be carefully evaluated in patients with impaired renal function, during long-term treatment, and when using delivery systems designed to increase systemic or tissue exposure.

Regarding potential drug–drug interactions, animal studies have shown that HSYA can alter the activity and mRNA expression of several cytochrome P450 enzymes, with inhibitory effects reported particularly for CYP1A2 and CYP2C11. Given its antiplatelet activity, microcirculation-enhancing effects, and influence on coagulation parameters [39], concomitant administration of HSYA with anticoagulants, antiplatelet agents, or drugs metabolized by susceptible CYP isoforms may increase the risk of pharmacokinetic interactions or bleeding and should therefore be approached cautiously.

From an ophthalmic translational perspective, evidence supporting the safety of systemic HSYA administration cannot be directly extrapolated to local ocular use. The development of eye drops, intraocular injections, nanocarrier-based systems, or sustained-release formulations will require dedicated assessment of corneal and conjunctival irritation, cytotoxicity in retinal pigment epithelial cells and retinal neurons, changes in intraocular pressure, electroretinographic function, fundus histopathology, and inflammatory responses following prolonged local exposure. Overall, HSYA has a comparatively favorable preclinical and clinical safety foundation; however, its ophthalmic development will require systematic evaluation of local toxicity and long-term ocular safety.

## 4. Evidence for HSYA in Diabetic Retinopathy and Related Retinal Models: Mechanistic Interpretation

The potential effects of HSYA in oxidative stress-related retinal diseases involve a network of interconnected pathological processes, including impaired antioxidant defense, amplification of inflammatory signaling, mitochondrial dysfunction, disruption of the neurovascular unit, and regulated cell death. However, the ophthalmic relevance and mechanistic certainty of the available evidence vary considerably. Direct ocular evidence is currently derived primarily from models of diabetic retinopathy, whereas studies of retinal photic injury remain limited. The proposed effects of HSYA in retinal ischemia–reperfusion injury, glaucoma, and age-related macular degeneration are inferred largely from non-ocular models or from shared pathological mechanisms. In view of these limitations, this section critically evaluates the potential retinal protective effects of HSYA according to disease model and level of evidence.

### 4.1. Diabetic Retinopathy: Direct Ocular Evidence and Mechanistic Interpretation

Diabetic retinopathy currently represents the ocular disease model in which evidence for HSYA is most concentrated. Rather than being solely a microvascular disorder, DR is increasingly recognized as a disease of retinal neurovascular unit dysfunction involving retinal neurons, Müller glia, microglia, vascular endothelial cells, and pericytes. Sustained hyperglycemia promotes reactive oxygen species (ROS) generation through several interconnected mechanisms, including disruption of the mitochondrial electron transport chain, formation of advanced glycation end products and activation of AGE–RAGE signaling, stimulation of protein kinase C pathways, and activation of NADPH oxidases. In parallel, reduced activities of antioxidant enzymes, including superoxide dismutase, catalase, and glutathione peroxidase, together with glutathione depletion, weaken endogenous antioxidant defenses and promote the accumulation of lipid peroxidation products such as malondialdehyde [40,41,42]. Excessive ROS subsequently contributes to neuronal and endothelial injury, blood–retinal barrier disruption, increased vascular permeability, and VEGF-associated vascular responses, thereby linking metabolic dysregulation to inflammation and microcirculatory dysfunction.

Direct evidence is currently derived primarily from animal models of diabetic retinopathy. Following HSYA treatment, retinal histopathological abnormalities and blood–retinal barrier disruption were attenuated, accompanied by reductions in the apoptotic index of the retinal ganglion cell layer and in the levels of IL-1β, TNF-α, and malondialdehyde. Superoxide dismutase activity was increased, together with the expression of Nrf2, HO-1, and Bcl-2 [31]. These findings indicate that systemic administration of HSYA is associated with improvements in retinal oxidative stress, inflammation, and apoptosis, suggesting that its effects may extend beyond direct free-radical scavenging.

Activation of the Nrf2-dependent antioxidant response may contribute to these effects. Under basal conditions, Keap1 binds to the amino-terminal Neh2 domain of Nrf2 and represses antioxidant response element-dependent transcription [43]. Under oxidative stress, Nrf2 stabilization and nuclear accumulation promote the expression of antioxidant-response genes, including HO-1, NQO1, and genes involved in glutathione metabolism [44]. In diabetic retinopathy, Nrf2 signaling also intersects with inflammatory pathways, mitochondrial homeostasis, autophagy, ferroptosis, and vascular responses [44]. The increased expression of Nrf2 and HO-1 observed after HSYA treatment is therefore consistent with the accompanying improvement in retinal antioxidant indices [31]. However, existing studies have not used pharmacological inhibition, gene silencing, or genetic deletion to establish whether Nrf2 is required for the retinal protective effects of HSYA. The available findings should thus be interpreted as associative rather than definitive mechanistic evidence.

Studies of HSYA in non-ocular ischemic models provide indirect support for this mechanistic interpretation. A systematic review and meta-analysis of animal studies showed that HSYA reduced malondialdehyde levels, increased superoxide dismutase activity, nitric oxide levels, and endothelial nitric oxide synthase activity, and upregulated Nrf2 mRNA expression [17]. These findings indicate that HSYA can modulate indices associated with oxidative stress and endothelial function. However, substantial differences between cardiac and retinal tissues in architecture, local drug exposure, and target-cell composition preclude the use of these findings as retina-specific validation.

Reciprocal amplification between oxidative stress and inflammation may contribute to the integrated effects of HSYA in diabetic retinopathy. Reactive oxygen species can activate redox-sensitive transcription factors, including NF-κB and AP-1, and stimulate stress-responsive pathways such as p38 MAPK, thereby promoting the expression of TNF-α, IL-1β, IL-6, MCP-1, and adhesion molecules. Activated microglia, reactive Müller glia, infiltrating leukocytes, and injured endothelial cells can, in turn, release additional reactive oxygen species and pro-inflammatory mediators, further exacerbating endothelial dysfunction, blood–retinal barrier disruption, and neuronal injury [6,14,45,46]. Hyperglycemia, mitochondrial damage, and reactive oxygen species accumulation may also promote activation of the NLRP3 inflammasome, leading to caspase-1 activation and the maturation and release of IL-1β and IL-18 [47].

The reductions in IL-1β, TNF-α, and oxidative stress markers observed after HSYA treatment suggest that HSYA may attenuate the reciprocal amplification between oxidative stress and inflammation [31]. In experimental models of central nervous system injury, HSYA has been reported to reduce neuroinflammation and modulate microglial phenotypes through SIRT1/HMGB1/NF-κB-related signaling [48]. Other studies suggest that it may regulate microglial polarization and barrier injury through mechanisms involving the PI3K/Akt/mTOR pathway [20]. Because the blood–brain barrier and blood–retinal barrier share certain structural features, including endothelial tight junctions and glial support, these findings provide plausible mechanistic leads for retinal research. The p38 MAPK pathway is an important stress-responsive node in high-glucose-induced retinal injury, and inhibition of this pathway has been reported to attenuate inflammatory responses in retinal cells and diabetic animals [49,50]. However, whether HSYA directly regulates p38 MAPK signaling in the retina remains unknown.

Mitochondrial dysfunction and apoptosis are major downstream consequences of sustained oxidative stress and inflammatory activation. Hyperglycemic conditions can impair mitochondrial membrane potential and electron transport chain function, reduce ATP production, and promote the accumulation of mitochondrial reactive oxygen species and mitochondrial DNA damage. These events may subsequently trigger the opening of the mitochondrial permeability transition pore, release of cytochrome c, and activation of the caspase cascade. In the available diabetic retinopathy study, HSYA treatment increased Bcl-2 expression, reduced p53 expression, and improved indices of apoptosis [31]. These changes associate HSYA treatment with reduced retinal cell apoptosis but do not establish the involvement of the intrinsic mitochondrial apoptotic pathway. Key mechanistic endpoints, including mitochondrial membrane potential, mitochondrial permeability transition pore opening, cytosolic cytochrome c release, the Bax/Bcl-2 ratio, and caspase-9 activation, have not yet been systematically evaluated.

Overall, diabetic retinopathy models currently provide the most direct evidence that HSYA can influence retinal outcomes; however, the available studies remain at the preclinical proof-of-concept stage. No clear relationship has yet been established between ocular tissue exposure and pharmacodynamic effects, and evidence regarding dose–response relationships, long-term safety, comparative routes of administration, and functional outcomes assessed by electroretinography, optical coherence tomography, or retinal blood-flow measurements remains lacking. HSYA can therefore be considered to have shown preliminary retinal protective activity in experimental diabetic retinopathy, but the current evidence is insufficient to establish a definitive disease-modifying effect.

### 4.2. Retinal Photic Injury and Ischemia–Reperfusion Injury: Limited Ocular Evidence and Mechanistic Extrapolation

Retinal photic injury and ischemia–reperfusion injury arise from distinct initiating events but converge during disease progression on several pathological processes, including redox imbalance, mitochondrial dysfunction, lipid peroxidation, inflammation, and disruption of the blood–retinal barrier. Cellular susceptibility to photic injury depends on the wavelength, irradiation dose, and duration of light exposure, with retinal pigment epithelial cells and photoreceptors generally representing the principal cellular targets. By contrast, acute retinal ischemia initially affects the metabolically demanding inner retina, in which retinal ganglion cells (RGCs) and glial cells are particularly vulnerable. This may be followed by microvascular endothelial injury, pericyte dysfunction, and increased blood–retinal barrier permeability. Because both forms of injury involve disruption of mitochondrial homeostasis and retinal neurovascular unit integrity, they provide complementary experimental settings in which to investigate the potential retinal protective effects of HSYA.

Studies of light-induced retinal injury have shown that excessive light exposure induces oxidative stress, mitochondrial dysfunction, and apoptosis in photoreceptors and retinal pigment epithelial cells. Caspase-3 and other caspase-dependent apoptotic processes contribute to photoreceptor loss under these conditions [51,52]. Direct ocular evidence for HSYA in retinal photic injury is currently limited to one published animal study. Available findings suggest that HSYA may attenuate light-induced retinal histopathological injury and apoptosis-related changes, including caspase-3-associated responses. However, the evidence remains preliminary because the number of studies is small, experimental light conditions and dosing regimens are not standardized, and ocular pharmacokinetics, optical coherence tomography, electroretinography, and visually guided behavioral outcomes have not been systematically evaluated. These findings therefore support further disease-specific investigation but do not establish a reproducible functional benefit. Future studies should use standardized light-injury models involving retinal pigment epithelial cells, photoreceptors, or experimental animals, with explicit reporting of light wavelength, irradiance, exposure duration, HSYA dose, and treatment timing. Retinal morphology, caspase-associated apoptosis, oxidative stress indices, and functional outcomes assessed by electroretinography or visually guided behavior should be evaluated in parallel.

Compared with retinal photic injury, direct evidence for HSYA in retinal ischemia–reperfusion models is even more limited, and its potential mechanisms are inferred predominantly from studies of cerebral ischemia–reperfusion and other non-ocular neurovascular injuries. In experimental cerebral ischemia, HSYA has been reported to inhibit pathological opening of the mitochondrial permeability transition pore (mPTP), reduce reactive oxygen species generation, and limit cytochrome c release, potentially through regulation of MEK/ERK/CypD signaling [53]. Other studies suggest that HSYA can attenuate oxidative injury and apoptosis while preserving tissue barrier integrity after ischemia–reperfusion [54]. Because both the retina and brain exhibit high oxygen demand and substantial dependence on mitochondrial metabolism, these findings provide mechanistic leads for retinal investigation. Nevertheless, the two tissues differ in cellular composition, local circulatory regulation, and barrier organization. Mechanisms demonstrated in cerebral ischemia models, therefore, cannot be assumed to operate in retinal ischemia–reperfusion injury without direct experimental validation.

Studies in brain microvascular endothelial cells further suggest that HSYA can suppress HIF-1α/NOX2-related signaling, reduce ROS-mediated inflammatory injury, and preserve the expression of tight-junction proteins such as ZO-1 [32,33]. Because NOX2 activation and tight-junction disruption also contribute to blood–retinal barrier dysfunction, this mechanism may be relevant to the potential retinal vasoprotective effects of HSYA. However, brain and retinal microvascular endothelial cells differ in transporter profiles, regulation of junctional proteins, and interactions with pericytes and glial cells. These effects therefore require direct validation in retinal microvascular endothelial cells, retinal pigment epithelial cells, and co-culture systems incorporating pericytes or Müller glia. Barrier protection should be assessed using complementary endpoints, including transendothelial electrical resistance, tracer permeability, and the continuity and localization of tight-junction proteins.

In non-ocular ischemic models, HSYA has also been reported to promote endothelial cell proliferation, migration, and tube formation, thereby supporting microvascular repair in ischemic tissues [55]. However, revascularization in peripheral ischemic tissue cannot be equated with a beneficial vascular response in retinal disease. In the ischemic retina, where non-perfused regions coexist with sustained VEGF elevation, stimulation of endothelial proliferation could either facilitate physiological reperfusion or exacerbate pathological neovascularization and vascular leakage. Evaluation of HSYA-induced vascular remodeling in the ischemic retina must therefore distinguish reparative revascularization from aberrant neovascular growth. In addition to in vitro migration and tube-formation assays, future studies should quantify non-perfused retinal areas, capillary reperfusion, vascular leakage, and neovascular lesion area to determine the overall vascular effect of HSYA.

Dysregulated autophagy and ferroptosis may further contribute to retinal cell loss following photic injury and ischemia–reperfusion. Evidence from non-ocular models indicates that HSYA can modulate neuronal autophagy and suppress NLRP3 inflammasome activation after acute traumatic brain injury [56]. In myocardial ischemia–reperfusion injury, HSYA has also been reported to suppress NLRP3 inflammasome activation and activate autophagy [57]. In neuronal injury models induced by oxygen–glucose deprivation/reoxygenation, HSYA or related bioactive constituents of safflower have also been reported to reduce iron accumulation, reactive oxygen species generation, and membrane lipid peroxidation, potentially through regulation of the system Xc^−^/GSH/GPX4 antioxidant axis [58]. These observations raise the possibility of reciprocal interactions among mitochondrial injury, impaired autophagic flux, inflammasome activation, and iron-dependent lipid peroxidation. However, there is currently insufficient evidence that HSYA directly regulates these processes in retinal pigment epithelial cells or photoreceptors exposed to photic injury, or in retinal ganglion cells subjected to ischemic stress. Autophagy and ferroptosis should therefore be regarded as candidate mechanisms requiring direct experimental validation rather than as established ocular pathways of HSYA action.

Future studies should evaluate histological protection, cell-specific mechanisms, and visual function within an integrated experimental framework. Mitochondrial and oxidative injury should be characterized using complementary endpoints, including reactive oxygen species generation, lipid peroxidation, mitochondrial membrane potential, ATP production, and mitochondrial permeability transition pore opening. Barrier and vascular outcomes should include tight-junction protein expression and localization, blood–retinal barrier permeability, retinal perfusion, and vascular leakage. At the cellular level, the survival and functional integrity of retinal ganglion cells, photoreceptors, and retinal pigment epithelial cells should be quantified separately. These findings should then be corroborated by integrated functional outcomes, including electroretinography, optical coherence tomography, and visually guided behavioral assessments.

In parallel, ocular pharmacokinetic studies should define the magnitude and temporal profile of HSYA exposure in the retina, choroid, vitreous, and aqueous humor following different routes of administration and establish exposure–response relationships between ocular tissue concentrations and pharmacodynamic outcomes. Mechanisms identified in non-ocular models can support the development of retinal therapeutic strategies only after pharmacologically effective exposure has been demonstrated in the relevant ocular target tissues.

### 4.3. Glaucoma and Age-Related Macular Degeneration: Mechanistic Relevance and Evidence Gaps

Glaucoma and AMD are both characterized by redox imbalance, mitochondrial dysfunction, and chronic low-grade inflammation; however, they differ fundamentally in their principal vulnerable cell populations, tissue microenvironments, and patterns of disease progression. The defining pathological feature of glaucoma is the progressive loss of retinal ganglion cells (RGCs) and their axons, accompanied by impaired axonal transport, remodeling of the optic nerve head, glial activation, and disruption of cellular energy metabolism. By contrast, AMD—particularly non-neovascular AMD—primarily affects the RPE–Bruch’s membrane–choroid complex and is characterized by loss of RPE homeostasis, secondary photoreceptor injury, lipid and protein deposition, complement dysregulation, and impairment of the outer blood–retinal barrier [59,60,61]. Although oxidative stress contributes to both diseases, the injury thresholds, metabolic dependencies, and microenvironmental signals of the affected cell populations differ substantially. Their potential responsiveness to HSYA, therefore, cannot be inferred solely from its general antioxidant activity.

The reported antioxidant, mitochondrial-protective, and anti-inflammatory activities of HSYA are mechanistically relevant to selected components of glaucomatous injury. In experimental models of cerebral ischemia and neuroinflammation, HSYA has been reported to attenuate mitochondrial damage, suppress apoptotic signaling, and limit inflammatory responses [48,53,54]. These findings raise the possibility that HSYA could influence RGC energy homeostasis, mitochondria-dependent apoptosis, and glia-mediated amplification of inflammation.

RGC degeneration, however, has disease-specific features that cannot be adequately represented by non-ocular neuroprotective models. The energetic support of the long RGC axon depends heavily on mitochondrial transport and function along the optic nerve, and disruption of axonal transport can initiate a degenerative cascade independently of oxidative stress within the neuronal soma. Protective effects observed for HSYA in non-ocular neurological models, therefore, cannot be directly extrapolated to these glaucoma-specific processes. To date, no study has systematically evaluated the effects of HSYA on RGC survival, axonal integrity, or optic nerve function in models of chronic ocular hypertension, optic nerve crush, or ischemic injury to the optic nerve head. Whether HSYA can delay or modify glaucomatous neurodegeneration, therefore, remains an open question.

In the context of AMD, the pharmacological profile of HSYA is likewise mechanistically relevant but remains far from constituting evidence of therapeutic efficacy. RPE cells are chronically exposed to high oxygen tension, photo-oxidative stress, a substantial phagocytic burden, and membranes enriched in polyunsaturated fatty acids, rendering them particularly susceptible to mitochondrial injury and lipid peroxidation [62,63]. Findings from non-AMD models indicating that HSYA may modulate Nrf2-driven antioxidant defenses, NADPH oxidase activity, glutathione homeostasis, and autophagic flux provide testable biological hypotheses for RPE protection [31,32,56,58].

However, the pathological spectrum of AMD extends well beyond oxidative injury. The age-related accumulation of lipofuscin and A2E, dysregulation of complement signaling, lipid deposition and thickening of Bruch’s membrane, impaired choroidal perfusion, and age-associated metabolic reprogramming collectively impose multiple chronic insults on the RPE–photoreceptor interface [63,64,65]. Conventional oxidative stress models reproduce only a limited subset of these pathological processes. Reductions in ROS or MDA levels may demonstrate antioxidant activity, but they are insufficient to support the conclusion that HSYA has disease-modifying potential in AMD.

For glaucoma, the immediate priority is to determine whether HSYA can confer meaningful protection to the RGC–axon unit under conditions of sustained intraocular pressure elevation or optic nerve axonal injury, rather than merely improving retinal oxidative stress markers. Experimental designs should therefore incorporate models of chronic ocular hypertension or optic nerve injury and evaluate a coordinated set of endpoints, including RGC survival, axonal integrity, optic nerve morphology, mitochondrial function, glial responses, and functional measures such as visual evoked potentials, electroretinography, and visually guided behavior [66,67,68].

AMD research, by contrast, must distinguish acute oxidative injury to the RPE from chronic age-related degeneration. A2E/blue-light exposure, sodium iodate-induced injury, and other RPE damage models may be useful for initial mechanistic screening, but their findings should subsequently be related to RPE polarity and barrier function, photoreceptor-supporting capacity, complement-associated inflammation, patterns of lipid deposition, and structural and functional changes in the outer retina. No single model can recapitulate the full pathological spectrum of AMD; robust conclusions will therefore require cross-validation across complementary experimental systems.

Overall, the proposed relevance of HSYA to glaucoma and AMD remains confined to shared pathological pathways and has not yet been supported by disease-specific experimental evidence. The first priority for future research is to determine whether different routes of administration can achieve measurable, pharmacologically relevant exposure to HSYA in RGCs, the optic nerve, the RPE, and the choroid–retina interface. These studies should establish quantitative relationships between tissue exposure and molecular, structural, and functional outcomes. Only after this pharmacokinetic and exposure–response foundation has been established will it be scientifically justified to assess whether the general antioxidant and anti-inflammatory activities of HSYA can translate into meaningful protection against glaucomatous neurodegeneration or AMD-associated degeneration of the outer retina.

### 4.4. Shared Mechanisms, Evidence Grading, and a Framework for Translational Validation

The integration of direct ocular studies, non-ocular neurovascular models, and the pathological mechanisms of different retinal diseases identifies several biological processes that may be relevant to the actions of HSYA. Across multiple experimental settings, recurrent findings involve Nrf2/HO-1-associated antioxidant responses, mitochondria-dependent apoptosis, NOX2-mediated oxidative stress, and preservation of tight-junction proteins [31,32,33,53]. These findings suggest that HSYA may act on interconnected regulatory nodes linking redox homeostasis, inflammation, mitochondrial function, and cell survival, rather than on a single disease-specific molecular target.

However, the recurrence of similar molecular changes across distinct experimental models does not establish that these pathways have the same causal role in the retina. In DR models, for example, increased Nrf2 and HO-1 expression following HSYA treatment has been associated with improvements in retinal histopathology and oxidative stress indices [31]. Nevertheless, no study has yet used pharmacological inhibition, gene silencing, genetic deletion, or cell-type-specific intervention to demonstrate that Nrf2 is required for the retinal protective effects of HSYA.

The principal limitation of the current evidence is not a lack of plausible candidate mechanisms but rather the limited amount of direct ophthalmic evidence and the absence of causal confirmation. Retina-specific data are derived mainly from animal models of diabetic retinopathy, in which the reported endpoints have focused on histopathology, blood–retinal barrier injury, and markers of oxidative stress, inflammation, and apoptosis [31]. Because functional assessments such as electroretinography, visual evoked potentials, and visually guided behavioral tests have not been incorporated, and in vivo structural measures such as optical coherence tomography are also lacking, it remains uncertain whether the observed molecular and histological changes translate into sustained visual benefit [31,69].

Evidence from retinal photic injury is limited to a small number of studies reporting histological and apoptosis-related outcomes, with substantial heterogeneity in light-exposure conditions, dosing regimens, and evaluation endpoints. Mechanistic hypotheses for retinal ischemia–reperfusion injury are supported primarily by studies of cerebral ischemia and microvascular endothelial injury, whereas the proposed relevance of HSYA to glaucoma and AMD is based largely on shared pathological processes. Disease-specific features, including impaired axonal transport, defective phagocytic function of the RPE, complement dysregulation, and Bruch’s membrane remodeling, have not yet been directly linked to HSYA treatment.

The ophthalmic translation of HSYA requires resolution of three interrelated questions. The first concerns ocular exposure: whether different routes of administration can achieve and sustain pharmacologically relevant concentrations of HSYA in the retina, RPE, optic nerve, or choroid–retina interface. Existing ocular studies have not provided sufficient pharmacokinetic data to establish a relationship between ocular tissue exposure and pharmacological response [31].

The second question concerns disease-relevant effects: once adequate target-tissue exposure has been confirmed, whether HSYA can reproducibly modify the key pathological processes represented by each disease model. These include blood–retinal barrier dysfunction in diabetic retinopathy [31], injury to the RPE and photoreceptors following excessive light exposure [51,52], and degeneration of the RGC–axon unit in glaucoma [66,67,68]. For retinal ischemia–reperfusion injury, current mechanistic hypotheses are derived mainly from cerebral ischemia–reperfusion studies rather than from direct retinal evidence [53,54]. Finally, it must be determined whether molecular and histological protection translates into improvements in functional outcomes, including electroretinography, visual evoked potentials, and visually guided behavior, and whether these functional changes are concordant with structural findings obtained by optical coherence tomography.

Ocular exposure, disease-relevant effects, and visual function form an interdependent translational evidence chain. If a given administration route fails to achieve pharmacologically relevant exposure in ocular target tissues, claims of direct ocular efficacy and local mechanism lack a sufficient pharmacokinetic basis [70,71]. Conversely, tissue detection of HSYA or isolated biomarker changes alone cannot establish a disease-modifying effect. Current evidence is concentrated mainly in animal models of diabetic retinopathy, whereas retinal photic injury, ischemia–reperfusion injury, glaucoma, and AMD remain insufficiently validated. HSYA should therefore be regarded as a candidate requiring further ocular pharmacokinetic and disease-specific evaluation, rather than as an established ophthalmic lead compound.

Future studies should be tailored to the maturity of evidence in each disease model. In diabetic retinopathy, priorities include dose–exposure–response analysis, causal validation of key pathways, and integration of structural and functional outcomes. Studies of retinal photic injury and ischemia–reperfusion should standardize experimental conditions and assess ocular exposure, cell-specific injury, and visual function. For glaucoma and AMD, early work should combine RGC- or RPE-based screening with route comparison and ocular distribution studies. The current evidence and key research gaps are summarized in Table 2.

## 5. Ocular Delivery Barriers and Target-Site Delivery Strategies

### 5.1. Systemic Pharmacokinetic Characteristics and Limitations of Systemic Exposure

HSYA is a quinochalcone C-glycoside characterized by high aqueous solubility and low lipophilicity. Its pronounced polarity facilitates the preparation of aqueous formulations but restricts passive transmembrane permeation and gastrointestinal absorption. HSYA has consequently been characterized as a high-solubility, low-permeability compound, with an oral bioavailability of approximately 1.2%. Following oral administration, its plasma concentration–time profile may exhibit a double-peak pattern, suggesting that the absorption, transport, and systemic disposition of HSYA involve multiple and potentially complex processes [72].

Studies using epithelial cell monolayers indicate that the intestinal absorption of HSYA may involve a combination of paracellular diffusion, transcellular transport, and carrier-mediated uptake and efflux [73]. Following oral administration, HSYA has been detected in highly perfused tissues, including the liver, kidneys, and lungs. However, the available distribution data do not demonstrate that HSYA achieves stable, pharmacologically relevant exposure in posterior ocular tissues [74]. Plasma concentrations or drug levels measured in major systemic organs, therefore, cannot be used to infer actual exposure in the retina.

HSYA undergoes multiple biotransformation reactions in vivo, including reduction, hydrolysis, hydroxylation, methylation, and glucuronidation. HSYA-related metabolites have been detected in plasma, bile, urine, and feces after oral administration [38]. However, these data do not establish a quantitatively dominant excretion route or define the ocular metabolism and elimination of HSYA. A study in healthy volunteers showed that intravenously administered HSYA was generally well tolerated, with a terminal elimination half-life of approximately 4.0–4.7 h and no evident accumulation after repeated dosing [34]. This relatively rapid systemic clearance may limit the maintenance of sustained exposure in ocular target tissues. Increasing the administered dose or dosing frequency to prolong exposure would also require careful assessment of systemic safety, as repeated-dose animal studies have identified coagulation abnormalities and renal toxicity as potential concerns.

Natural deep eutectic solvent systems have been investigated to improve the extraction efficiency and oral bioavailability of HSYA and related safflower yellow pigments [30,75], whereas nanoemulsion-based formulations have been explored to enhance intestinal absorption and systemic exposure [76]. These studies demonstrate that the pharmacokinetic behavior of HSYA can be modified to some extent through formulation design. However, enhanced gastrointestinal absorption or systemic bioavailability does not establish that HSYA can reach the retina at pharmacologically relevant concentrations. Current systemic pharmacokinetic data are therefore most useful for defining the absorption, distribution, metabolism, and elimination characteristics of HSYA, whereas its ophthalmic translation requires independent pharmacokinetic evidence from ocular tissues.

### 5.2. Ocular Barriers and Gaps in Ocular Tissue Pharmacokinetic Evidence for HSYA

Drug delivery to the posterior segment is constrained by multiple anatomical and physiological barriers. Following topical administration, tear dilution, blinking, and nasolacrimal drainage rapidly reduce precorneal drug residence [77]. Corneal epithelial tight junctions and conjunctival vascular clearance further limit ocular entry, while tissue diffusion, choroidal clearance, and the inner and outer blood–retinal barriers restrict posterior segment distribution [71,78]. Accordingly, increased exposure in the tear film, cornea, or aqueous humor does not necessarily translate into a corresponding increase in drug concentrations within the vitreous, neural retina, or retinal pigment epithelium–choroid complex [79].

In diabetic retinopathy, HSYA may act on multiple components of the retinal neurovascular unit, including structures associated with the outer blood–retinal barrier and the retinal pigment epithelium–choroid complex [80,81,82]. Potential cellular targets include retinal microvascular endothelial cells, pericytes, Müller glia, microglia, retinal ganglion cells, and retinal pigment epithelial cells. The inner blood–retinal barrier is formed primarily by retinal capillary endothelial cells and their intercellular tight junctions, whereas the outer barrier depends on junctional complexes between adjacent retinal pigment epithelial cells [31,82]. Together, these specialized barrier systems maintain retinal metabolic, ionic, and immune homeostasis. At the same time, they restrict the entry of highly polar compounds from the systemic circulation into the neural retina, thereby presenting a major obstacle to achieving pharmacologically relevant retinal exposure to HSYA.

The high polarity, low lipophilicity, and limited membrane permeability of HSYA create substantial uncertainty regarding its ability to cross cellular membranes and tight-junction barriers. Although the blood–brain barrier and blood–retinal barrier share certain structural features, they differ in endothelial phenotype, transporter expression, pericyte support, glial microenvironment, and regulation of local blood flow [70,83,84]. Evidence of drug distribution within the brain may therefore provide only limited mechanistic guidance and cannot be used to predict the ocular tissue distribution or retinal pharmacokinetics of HSYA.

Diabetes, ischemia, and inflammation can increase blood–retinal barrier permeability; however, such disruption is spatially and temporally heterogeneous and is frequently accompanied by retinal edema, inflammation, and tissue injury. Increased barrier permeability does not imply uniform drug penetration into all relevant target cells, nor does it ensure stable and reproducible exposure within ocular tissues. Moreover, the breakdown of the blood–retinal barrier is a central pathological feature of diabetic retinopathy and diabetic macular edema. Preservation or restoration of the structural and functional integrity of both the inner and outer blood–retinal barriers, therefore, represents an important therapeutic objective in diabetic retinopathy [15]. The ophthalmic development of HSYA should not rely on uncontrolled barrier leakage under pathological conditions. Pharmacologically relevant exposure in posterior ocular tissues must instead be confirmed through direct measurement of HSYA concentrations in the relevant ocular compartments.

### 5.3. Route-of-Administration-Based Strategies for Posterior Segment Drug Delivery

Different routes of administration expose a drug to distinct anatomical barriers, absorption pathways, and clearance mechanisms, thereby substantially affecting the magnitude and duration of exposure in the aqueous humor, vitreous, neural retina, and retinal pigment epithelium–choroid complex [71,85]. For HSYA, route selection should therefore be guided by target-tissue exposure, local residence time, systemic exposure, and administration-related safety, rather than by oral bioavailability or formulation characteristics alone.

Oral administration is convenient; however, HSYA must sequentially overcome gastrointestinal absorption, systemic distribution, and the blood–retinal barrier. Whether this route can achieve pharmacologically relevant concentrations in the posterior segment remains unknown. Intravenous administration bypasses gastrointestinal absorption, and human pharmacokinetic and short-term safety data are available for this route [34]. Nevertheless, existing studies have not measured HSYA concentrations in the aqueous humor, vitreous, retina, or retinal pigment epithelium–choroid complex. It is therefore not yet possible to determine whether intravenous administration is suitable for long-term intervention in diabetic retinopathy. Its feasibility will ultimately depend on the balance between ocular delivery efficiency and systemic safety.

Topical ocular administration is non-invasive, readily repeatable, and potentially better suited to long-term treatment. However, precorneal clearance and the corneal epithelial barrier substantially limit the fraction of an administered dose that enters the eye. In selected drug–model combinations, chitosan-modified liposomes and deformable liposomes have shown potential to improve intraocular or posterior segment distribution [86]. Such benefits are highly dependent on the physicochemical properties of the drug, carrier composition, and experimental model and therefore cannot be directly extrapolated to HSYA. The evaluation of topical HSYA formulations should focus on whether they produce a measurable increase in exposure within posterior ocular tissues. Prolonged ocular-surface residence, enhanced ex vivo corneal permeation, or increased aqueous humor concentrations alone would be insufficient to demonstrate effective delivery to the vitreous, neural retina, or retinal pigment epithelium–choroid complex.

Subconjunctival, periocular, and other transscleral routes can bypass part of the corneal barrier and reduce the diffusion distance between the administration site and the choroid–retina interface. Nevertheless, a drug must still traverse the sclera and choroid, withstand clearance by the local circulation, and cross the outer blood–retinal barrier before reaching retinal target tissues. Suprachoroidal administration places the drug in closer proximity to the choroid–retina interface, and previous studies have reported increased exposure within the retinal pigment epithelium–choroid–sclera complex. Clinical investigations of this route have also been conducted in posterior segment diseases [87]. However, the available evidence is derived predominantly from corticosteroids and remains insufficient to predict the distribution, retention, and clearance of highly hydrophilic HSYA following suprachoroidal administration.

Intravitreal injection can directly increase drug concentrations in the vitreous and shorten the diffusion distance to the retina. However, repeated injections impose a substantial treatment burden and are associated with cumulative procedure-related risks, including endophthalmitis [88]. Injectable microgels and thermosensitive hydrogels can provide sustained drug release and may therefore offer a formulation strategy for prolonging the intraocular residence of HSYA [89]. Before these delivery systems can be considered for HSYA, its stability, diffusion behavior, and clearance within the vitreous must be characterized, together with its local retinal toxicity and ocular tolerability.

Non-invasive routes are more amenable to repeated administration during long-term treatment but may provide insufficient delivery to the posterior segment. Invasive routes can achieve higher local drug exposure, although this advantage is accompanied by greater procedural burden and administration-related risks. The most appropriate route for HSYA, therefore, remains to be established through direct comparative studies integrating ocular tissue pharmacokinetics, duration of pharmacological activity, and local and systemic safety.

### 5.4. Ocular Delivery of Flavonoids and Related Glycosides: Implications for HSYA

Natural bioactive compounds that, like HSYA, contain glycosidic moieties and are limited by poor membrane permeability or short ocular-surface residence may provide useful methodological guidance for the development of ophthalmic HSYA formulations. However, substantial differences among these compounds in glycosidic linkage, sugar composition, molecular weight, ionization behavior, and lipid–water partitioning can markedly affect their ocular disposition. Evidence from related glycosides is therefore more appropriate for identifying candidate delivery systems and experimental evaluation strategies than for directly predicting the intraocular distribution of HSYA.

Puerarin is an isoflavone C-glycoside, whereas HSYA is a quinochalcone C-glycoside. Although they belong to different chemical subclasses, both contain relatively stable C-glycosidic linkages and exhibit high polarity and limited membrane permeability, which present broadly similar formulation challenges. In a recent study, puerarin was encapsulated in human serum albumin nanoparticles that were subsequently incorporated into a thermosensitive in situ gel [90]. This system prolonged drug release, increased ex vivo corneal flux and permeability coefficients, and extended the duration of the intraocular pressure-lowering effect in an animal model of chronic ocular hypertension. Nevertheless, the study focused primarily on in vitro drug release, corneal permeation, ocular tolerability, and local pharmacodynamic effects; drug concentrations in the vitreous, neural retina, and retinal pigment epithelium–choroid complex were not determined.

Studies of flavonoid O-glycosides, including rutin, hesperidin, and naringin, have further broadened the range of candidate delivery systems relevant to ophthalmic formulation development. Chitosan-coated PLGA nanoparticles co-loaded with forskolin and rutin exhibited favorable mucoadhesive properties and enhanced the ex vivo corneal permeation of both compounds [91]. Hesperidin was complexed with cyclodextrin and subsequently incorporated into a thermosensitive in situ gel, resulting in improved solubility, gelation properties, and ocular-surface tolerability [92]. An in situ gel formulation of naringin likewise showed an appropriate gelation temperature and viscosity, together with sustained in vitro drug release [93].

Collectively, these studies indicate that the local delivery performance of ophthalmic formulations is governed not only by particle size but also by surface charge, drug–carrier interactions, mucoadhesion, gelation behavior, and release kinetics. Nevertheless, most investigations have focused primarily on formulation characterization, ex vivo corneal permeation, and ocular-surface tolerability, with limited assessment of drug exposure in posterior ocular tissues.

Baicalin is a flavonoid O-glycoside. In a model of experimental autoimmune uveitis, a liposome-loaded thermosensitive hydrogel formulation of baicalin provided sustained drug release and attenuated intraocular inflammation, oxidative stress, and retinal structural damage [94]. This study extended the evaluation of ocular formulations beyond the ocular surface and cornea to include retinal histopathology and inflammatory endpoints. However, concentration–time profiles of baicalin in the vitreous, neural retina, and retinal pigment epithelium–choroid complex were not established. Improvements in retinal morphology and inflammatory markers provide evidence of ocular pharmacological activity but are insufficient to determine the actual magnitude and duration of retinal drug exposure or to establish a quantitative relationship between tissue exposure and pharmacodynamic response.

Available studies support the consideration of albumin- or polymer-based nanoparticles, lipid carriers, cyclodextrin complexes, and nanocarrier–hydrogel composite systems as candidate platforms for ocular delivery of HSYA. These studies provide useful guidance for carrier selection, formulation optimization, and safety assessment, but they do not resolve the central question of whether HSYA can achieve pharmacologically relevant exposure in the posterior segment. Any practical advantage of these candidate formulations must therefore be confirmed through direct comparative pharmacokinetic studies of free HSYA and carrier-based formulations in the vitreous, neural retina, and retinal pigment epithelium–choroid complex.

### 5.5. Pharmacokinetic—Pharmacodynamic Validation Requirements for HSYA Delivery Systems

At present, the principal evidence gap in the ophthalmic development of HSYA extends beyond incomplete mechanistic characterization to the absence of a direct link between drug exposure and retinal response. Existing studies in diabetic retinopathy models have reported attenuation of retinal histopathological abnormalities and blood–retinal barrier-associated injury, accompanied by changes in inflammatory and oxidative stress markers. However, HSYA concentrations were not measured concurrently in plasma, vitreous, neural retina, or the retinal pigment epithelium–choroid complex [31]. The observed retinal effects may reflect direct pharmacological activity following ocular tissue exposure, but they may also be influenced by systemic anti-inflammatory, metabolic, or vasoprotective actions. Retinal improvement after systemic administration alone therefore does not establish that HSYA has achieved pharmacologically relevant exposure in the posterior segment.

Candidate formulations must first possess quality attributes appropriate for the intended route of administration. Parameters such as particle size and size distribution, surface charge, encapsulation efficiency, drug loading, physical stability, release behavior, osmolality, pH, and sterility are essential for assessing manufacturability, storage stability, and batch-to-batch consistency [95,96]. These characteristics, however, do not in themselves demonstrate successful delivery to the posterior segment. Evidence that a formulation improves posterior ocular delivery requires direct comparison of the concentration–time profiles of free HSYA and its carrier-based formulation in the vitreous, neural retina, and retinal pigment epithelium–choroid complex. Such studies should also determine whether enhanced local exposure is accompanied by increased systemic exposure or greater distribution to non-target tissues.

Ocular tissue pharmacokinetic studies should, at a minimum, determine fundamental parameters such as the maximum concentration (C_max_), time to maximum concentration (T_max_), area under the concentration–time curve (AUC), and elimination half-life [97,98]. A single tissue concentration measured at a terminal time point cannot reliably distinguish transient drug penetration from sustained retention or local accumulation and is insufficient to establish a robust exposure–response relationship. For sustained-release and nanocarrier-based systems, additional characterization should address the initial burst release, drug liberation from the carrier, diffusion through the vitreous, and clearance from ocular tissues. Wherever analytically feasible, free or released HSYA should be distinguished from carrier-associated drugs to avoid counting unreleased HSYA within the formulation as pharmacologically available exposure at the target site.

Total ocular tissue concentration does not necessarily reflect pharmacologically active free drug exposure because binding within ocular matrices can reduce the unbound fraction [99]. Nominally effective concentrations identified in vitro should not be treated as in vivo retinal therapeutic thresholds without ocular pharmacokinetic–pharmacodynamic bridging. Where necessary, measurements of unbound or intracellular HSYA and spatially resolved analytical methods, including imaging mass spectrometry, should be used to confirm delivery to the intended ocular compartment [100].

In DR models, increases in Nrf2, HO-1, and Bcl-2 expression, together with decreased p53 expression and modulation of IL-1β, TNF-α, MDA, and SOD levels, have been reported following HSYA treatment [31]. These endpoints may serve as candidate pharmacodynamic readouts for future PK–PD studies; however, current evidence remains correlative.

Evidence that HSYA regulates NF-κB/MAPK signaling is derived mainly from a non-ocular osteoarthritis model [101], whereas inhibition of NLRP3 inflammasome activation has been demonstrated in LPS-stimulated RAW264.7 macrophages [102]. Ferroptosis-related effects of HSYA have been reported in myocardial ischemia–reperfusion models [103], while the ophthalmic relevance of ferroptosis is currently supported mainly by disease-mechanism literature [104]. These studies generate testable hypotheses but do not establish direct retinal target engagement by HSYA. Mechanistic validation should be aligned with ocular tissue exposure assessments, using matched dose groups and sampling time points to determine whether molecular changes occur after HSYA reaches the target tissue and whether they scale with exposure levels.

Pharmacokinetic and pharmacodynamic endpoints should be aligned across both dose levels and sampling times. Blood–retinal barrier permeability, vascular leakage, and histopathology can be used to assess local tissue injury, whereas optical coherence tomography provides in vivo measures of retinal thickness and structural integrity. Electroretinography, visual evoked potentials, and visually guided behavioral tests are required to determine whether these changes translate into functional benefit [69,105]. Convincing PK–PD evidence should demonstrate concordant dose–response and temporal relationships among ocular tissue exposure, molecular responses, retinal structural preservation, and visual function. Improvement in a single terminal endpoint, particularly an isolated change in an oxidative stress or inflammatory marker, is insufficient to establish a sustained local therapeutic effect.

Safety assessments should distinguish the effects of free HSYA, blank carriers, drug-loaded formulations, and the administration procedure itself. For topical formulations, key endpoints include ocular-surface irritation, corneal integrity, tear production, and long-term tolerability. Intravitreal, suprachoroidal, and other locally injected formulations additionally require evaluation of intraocular pressure, intraocular inflammation, vitreous clarity, retinal and RPE toxicity, and long-term visual function [89,105]. Sustained-release and nanocarrier systems may introduce further risks, including carrier deposition, particle aggregation, accumulation of degradation products, and toxicity associated with repeated administration. Systemic exposure should also be monitored, together with potential coagulation-related and renal safety signals.

**Figure 2:**
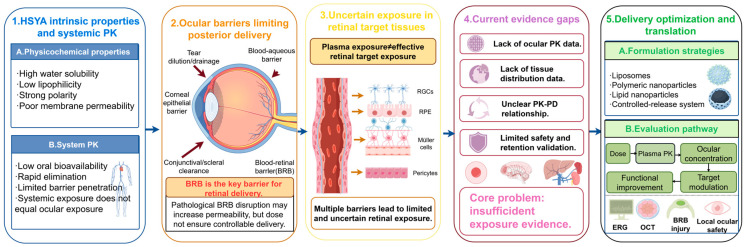
Pharmacokinetic and delivery barriers limiting ophthalmic translation of HSYA, including low membrane permeability, uncertain ocular tissue exposure, and the need for exposure-response validation. (https://www.figdraw.com/static/index.html#/, accessed on 30 June 2026).

The feasibility of ocular delivery of HSYA ultimately depends on establishing dose- and time-concordant exposure–response relationships linking target-tissue exposure to local pharmacological activity, preservation of retinal structure, improvement in visual function, and acceptable safety. Until such an evidence chain is established, formulation characteristics such as particle size, encapsulation efficiency, and sustained-release behavior, increases in systemic drug concentrations, or improvements in a single oxidative stress marker cannot independently support the ophthalmic translation of HSYA. The principal pharmacokinetic, anatomical, and delivery barriers limiting the ophthalmic translation of HSYA are summarized in Figure 2.

## 6. Conclusions

HSYA is a structurally defined, water-soluble quinochalcone C-glycoside derived from safflower. Available pharmacological studies provide a biological rationale for investigating HSYA in oxidative stress-related retinal injury, but mechanistic relevance alone does not constitute disease-specific ophthalmic evidence. Direct ocular evidence is currently derived mainly from animal models of diabetic retinopathy, in which HSYA may attenuate retinal tissue injury, blood–retinal barrier dysfunction and associated inflammatory and cellular damage. Direct evidence for retinal photic injury remains limited, whereas its potential effects in retinal ischemia–reperfusion injury, glaucoma, and age-related macular degeneration are still inferred largely from non-ocular neurovascular models and shared pathological processes. The available evidence is therefore insufficient to demonstrate that HSYA produces consistent, reproducible retinal protection or improves visual function across these conditions.

The central challenge in the ophthalmic translation of HSYA is whether different routes of administration can achieve and sustain pharmacologically relevant exposure in target tissues, including the retina, retinal pigment epithelium, choroid, and optic nerve. Its high hydrophilicity, limited membrane permeability, low oral bioavailability, and incompletely characterized ocular distribution may restrict delivery to the posterior segment. Future studies should compare ocular tissue distribution across administration routes, establish quantitative dose–exposure–response relationships, and concurrently evaluate disease-specific structural outcomes, visual function, and long-term ocular safety. Until reproducible ocular exposure–response relationships and improvements in visual function have been demonstrated, HSYA should be regarded as a candidate molecule requiring systematic ophthalmic validation rather than as an agent with established therapeutic value in retinal disease.

## Figures and Tables

**Table 1:** Physicochemical, Pharmacokinetic, and Safety Profiles of HSYA: Implications for Ophthalmic Translation.

Category	HSYA Features	Implications for Ophthalmic Translation
Chemical type	Quinochalcone C-glycoside	Representative bioactive constituent of safflower
Molecular weight	Approximately 612.5 g/mol	Large molecular size may limit barrier penetration
Solubility	High water solubility and low lipophilicity	Favorable for aqueous formulations but unfavorable for passive transmembrane diffusion
Stability	Sensitive to pH, light, temperature, and alkaline conditions	Formulation development should control degradation and protect HSYA from light-, heat-, and pH-induced instability
Oral bioavailability	Low	Oral administration alone may be insufficient to achieve effective posterior ocular exposure
Distribution profile	Systemic tissue distribution has been studied, but ocular tissue distribution remains insufficiently characterized	Ocular tissue pharmacokinetics need to be validated
Metabolism and excretion	HSYA-related metabolites have been detected in plasma, bile, urine, and feces, but the quantitatively dominant excretion route remains incompletely defined	Ocular metabolism and elimination require direct characterization
Safety	Generally well tolerated, but coagulation and renal safety require attention	Local ocular safety requires separate evaluation

**Table 2:** Direct ocular evidence and related indirect mechanistic models of HSYA.

Evidence Level	Research Focus	Model and Dose	Ocular-Relevant Mechanisms/Endpoints	Interpretation and Translational Gap
Direct ocular evidence	Diabetic Retinopathy [31]	STZ-induced diabetic rats; 30 mg/kg	Nrf2/HO-1 signaling, Bcl-2/p53-related apoptosis markers, IL-1β, TNF-α, MDA, SOD, BRB injury, and retinal ganglion cell apoptosis	HSYA shows direct retinal protection in DR, but dose–response data, ocular pharmacokinetics, long-term safety, ERG, and OCT endpoints remain lacking.
Ocular-relevant indirect evidence	Endothelial barrier and tight-junction Protection [32]	Brain microvascular endothelial cells under hypoxia/reoxygenation or related injury; 10–50 μM	HIF-1α/NOX2/ROS axis, ZO-1, endothelial barrier integrity	HSYA supports a BRB-protective hypothesis, but validation in retinal endothelial cells and BRB models is still required.
Ocular-relevant indirect evidence	Neurovascular injury in cerebral ischemia–reperfusion [48,53,54]	MCAO or cerebral ischemia–reperfusion animal models; 8–32 mg/kg	ROS, inflammatory mediators, vascular permeability, neurological function, SIRT1/HMGB1/NF-κB pathway	HSYA shows neurovascular protection in cerebral I/R models, but this cannot be directly extrapolated to retinal I/R injury.
Ocular-relevant indirect evidence	Mitochondrial injury and Apoptosis [53]	Cerebral ischemia–reperfusion or other non-ocular ischemic models; 50 mg/kg	mPTP opening, CypD, cytochrome c release, caspase cascade, mitochondrial membrane potential	HSYA may inhibit mPTP-mediated mitochondrial apoptosis, but direct evidence in retinal cells is still insufficient.
Ocular-relevant indirect evidence	Ferroptosis and Oxidative neuronal injury [58]	PC12 cell OGD/R model; 10–50 μM	System Xc^−^/GSH/GPX4 axis, ROS, iron accumulation, lipid peroxidation	HSYA may suppress ferroptosis-related oxidative injury, but validation in RGCs, RPE cells, and retinal models is needed.
Ocular-relevant indirect evidence	Neuroinflammation modulation [20,48]	LPS- or MCAO/R-induced central neuroinflammation models; 10–50 μM	SIRT1/HMGB1/NF-κB signaling, microglial polarization, TNF-α, IL-1β, inflammatory markers	HSYA modulates central neuroinflammation, but its effects on retinal microglia and Müller cell-mediated inflammation remain unclear.
Ocular-relevant indirect evidence	Systemic vascular inflammation and oxidative Stress [27]	ApoE^−^/^−^ mouse model of atherosclerosis; 30 mg/kg	SphK1/S1P/S1PR3, RhoA/ROCK, inflammation, oxidative stress	HSYA supports a broad vasoprotective rationale, but atherosclerosis models provide only distant indirect evidence for retinal disease.
Ocular-relevant indirect evidence	Myocardial ischemia–reperfusion injury [57]	Rat myocardial I/R model; 8–32 mg/kg	JAK2/STAT1, NLRP3 inflammasome, autophagy, mitochondrial energy metabolism	HSYA regulates oxidative stress, inflammation, autophagy, and mitochondria in myocardial I/R, but not retinal I/R directly.
Ocular-relevant indirect evidence	Systemic pharmacokinetics and delivery Optimization [29,30,34,38,72,73,74,75]	Non-ocular pharmacokinetic and formulation studies, including oral administration, intravenous administration, nanoemulsions, and NaDES-based systems	Bioavailability, membrane permeability, tissue exposure, stability, and formulation optimization	Formulation strategies may Improve HSYA exposure, but ocular PK data in the retina, vitreous, and RPE/choroid remain lacking.

Direct ocular evidence is currently limited mainly to diabetic retinopathy and retinal photic injury models. The remaining studies should be interpreted as ocular-relevant mechanistic evidence rather than direct evidence of retinal efficacy.

## Data Availability

No new data were created or analyzed in this study. Data sharing is not applicable to this article.

## References

[1] You L., Lin Y., Zheng Y., Han Z., Zeng L., Chen H. (2024). The impact of aging on ocular diseases: Unveiling complex interactions. Aging Dis..

[2] Cvekl A., Vijg J. (2024). Aging of the eye: Lessons from cataracts and age-related macular degeneration. Ageing Res. Rev..

[3] Zhang Y., Huang S., Xie B., Zhong Y. (2024). Aging, cellular senescence, and glaucoma. Aging Dis..

[4] Wiciński M., Fajkiel-Madajczyk A., Kurant Z., Rzepiński Ł., Słupski M. (2026). Review of Therapeutic Potential of Coenzyme Q10 in Ophthalmology: Focus on Age-Related Macular Degeneration, Glaucoma, and Retinitis Pigmentosa. Antioxidants.

[5] Xu X., Pang Y., Fan X. (2025). Mitochondria in oxidative stress, inflammation and aging: From mechanisms to therapeutic advances. Signal Transduct. Target. Ther..

[6] Abukhaled Y. (2025). Reactive oxygen species and oxidative stress in ocular disease: From molecular mechanisms to targeted therapies. Med. Hypothesis Discov. Innov. Ophthalmol..

[7] Yan Y., Wu Y., Zhao Y., Yang Y., An G., Liu Z., Qi D. (2025). A review on eye diseases induced by blue light: Pathology, model, active ingredients and mechanisms. Front. Pharmacol..

[8] Zhang Z., Shan X., Li S., Chang J., Zhang Z., Dong Y., Wang L., Liang F. (2024). Retinal light damage: From mechanisms to protective strategies. Surv. Ophthalmol..

[9] Kowluru R.A., Chan P.-S. (2007). Oxidative stress and diabetic retinopathy. J. Diabetes Res..

[10] Fernández-Albarral J.A., Ramírez A.I., de Hoz R., Matamoros J.A., Salobrar-García E., Elvira-Hurtado L., López-Cuenca I., Sánchez-Puebla L., Salazar J.J., Ramírez J.M. (2024). Glaucoma: From pathogenic mechanisms to retinal glial cell response to damage. Front. Cell. Neurosci..

[11] Reddy S.K., Devi V., Seetharaman A.T., Shailaja S., Bhat K.M., Gangaraju R., Upadhya D. (2024). Cell and molecular targeted therapies for diabetic retinopathy. Front. Endocrinol..

[12] Medina-Arellano A.E., Albert-Garay J.S., Medina-Sánchez T., Fonseca K.H., Ruiz-Cruz M., Ochoa-de la Paz L. (2024). Müller cells and retinal angiogenesis: Critical regulators in health and disease. Front. Cell. Neurosci..

[13] Shen W. (2025). Retinal neurovascular coupling: From mechanisms to a diagnostic window into brain disorders. Cells.

[14] Bai Y., Wang X., Qi F., Zuo X., Zou G. (2025). Mechanisms of action of retinal microglia in diabetic retinopathy. Int. J. Mol. Med..

[15] Li M., Yang L., Zhai H., Qiao L., Wang Z., An X., Wang J. (2025). A new perspective on protecting the blood-retinal barrier against injury in diabetic retinopathy: Mitophagy. Front. Endocrinol..

[16] Wang Y., An J., Zhou J., Chang L., Zhang Q., Peng F. (2025). Hydroxysafflor yellow A: A natural pigment with potential anticancer therapeutic effect. Front. Pharmacol..

[17] Mao T., Jiang K., Pang Y., Pan Y., Jia W., Gao Q., Lin Q. (2025). Hydroxysafflor yellow A for ischemic heart diseases: A systematic review and meta-analysis of animal experiments. Front. Pharmacol..

[18] Wang Q., Yang Z., Guo L., Li Z., Liu Y., Feng S., Wang Y. (2023). Chemical composition, pharmacology and pharmacokinetic studies of GuHong injection in the treatment of ischemic stroke. Front. Pharmacol..

[19] Bacchetti T., Morresi C., Bellachioma L., Ferretti G. (2020). Antioxidant and pro-oxidant properties of *Carthamus tinctorius*, hydroxy safflor yellow A, and safflor yellow A. Antioxidants.

[20] Zheng F., Guo X., Yan Q., Zhou Y., Hu E., Zhu H., Cheng M., Yu Z., Hu M., Ding R. (2025). Hydroxysafflor yellow A attenuates the blood-brain barrier dysfunction and neuroinflammation through anti-inflammatory microglial polarization after intracerebral hemorrhage. Neuropharmacology.

[21] Orgah J.O., He S., Wang Y., Jiang M., Wang Y., Orgah E.A., Duan Y., Zhao B., Zhang B., Han J. (2020). Pharmacological potential of the combination of *Salvia miltiorrhiza* (Danshen) and *Carthamus tinctorius* (Honghua) for diabetes mellitus and its cardiovascular complications. Pharmacol. Res..

[22] Xian B., Wang R., Jiang H., Zhou Y., Yan J., Huang X., Chen J., Wu Q., Chen C., Xi Z. (2022). Comprehensive review of two groups of flavonoids in *Carthamus tinctorius* L. Biomed. Pharmacother..

[23] Ren F., Tan Z., Hu S., Rao C., Xiang Q., Wen J., Chen Y., Peng C. (2025). Pharmacological actions and applications of safflower flavonoids. Front. Nutr..

[24] Bai H., Yang J., Wang R. (2025). *Carthamus tinctorius* L.: A comprehensive review of its ethnomedicine, phytochemistry, pharmacology, and clinical applications. Front. Pharmacol..

[25] Wang Z.-L., Wang H.-T., Chang G., Ye G., Zhang M., Chen J., Ye M. (2025). Elucidation of the biosynthetic pathway of hydroxysafflor yellow A. Nat. Commun..

[26] Wu Z., Li R., Sun M., Hu X., Xiao M., Hu Z., Jiao P., Pu S., Zhai J., Zhang J. (2024). Current advances of *Carthamus tinctorius* L.: A review of its application and molecular regulation of flavonoid biosynthesis. Med. Plant Biol..

[27] Xue X., Deng Y., Wang J., Zhou M., Liao L., Wang C., Peng C., Li Y. (2021). Hydroxysafflor yellow A, a natural compound from *Carthamus tinctorius* L with good effect of alleviating atherosclerosis. Phytomedicine.

[28] Zhu H., Ni X., Yu Z., Liang S., Qiu Y., Huang J., Tang T., Wang Y., Hu E., Li T. (2025). Emerging natural product development strategies drive the therapeutic potential exploration and clinical translation of hydroxysafflor yellow A. J. Pharm. Anal..

[29] Zhao F., Wang P., Jiao Y., Zhang X., Chen D., Xu H. (2020). Hydroxysafflor yellow A: A systematical review on botanical resources, physicochemical properties, drug delivery system, pharmacokinetics, and pharmacological effects. Front. Pharmacol..

[30] Tong X., Yang J., Zhao Y., Wan H., He Y., Zhang L., Wan H., Li C. (2021). Greener extraction process and enhanced in vivo bioavailability of bioactive components from *Carthamus tinctorius* L. by natural deep eutectic solvents. Food Chem..

[31] Sun Z., Wang Y., Xu R., Zhang S., Yang H., Song J., Chang T. (2022). Hydroxysafflor yellow A improved retinopathy via Nrf2/HO-1 pathway in rats. Open Life Sci..

[32] Li Y., Liu X., Zhang P., Li Y., Sun M., Wang Y., Wang S., Yang H., Liu B., Wang M. (2022). Hydroxysafflor yellow A blocks HIF-1α induction of NOX2 and protects ZO-1 protein in cerebral microvascular endothelium. Antioxidants.

[33] Hudson N., Campbell M. (2021). Tight junctions of the neurovascular unit. Front. Mol. Neurosci..

[34] Zhao X., Gu S., Wang X., Cai Y., Zhou Z. (2025). The Safety, Tolerability, and Pharmacokinetics of Active Ingredients From Hydroxysafflor Yellow A in Healthy Chinese Volunteers. Clin. Pharmacol. Drug Dev..

[35] Hu M.-z., Zhou Z.-y., Zhou Z.-y., Lu H., Gao M., Liu L.-m., Song H.-q., Lin A.-j., Wu Q.-m., Zhou H.-f. (2020). Effect and safety of hydroxysafflor yellow A for injection in patients with acute ischemic stroke of blood stasis syndrome: A phase II, multicenter, randomized, double-blind, multiple-dose, active-controlled clinical trial. Chin. J. Integr. Med..

[36] Liu Z., Li C., Li M., Li D., Liu K. (2004). The subchronic toxicity of hydroxysafflor yellow A of 90 days repeatedly intraperitoneal injections in rats. Toxicology.

[37] Qiu S., Li X., Wang X., Xiao P., Teng S., Wei G., Yang L., Liu Z., Mu Y. (2026). Developmental and reproductive toxicity studies of Hydroxysafflor Yellow a in Sprague-Dawley rats. Drug Chem. Toxicol..

[38] Jin Y., Wu L., Tang Y., Cao Y., Li S., Shen J., Yue S., Qu C., Shan C., Cui X. (2016). UFLC-Q-TOF/MS based screening and identification of the metabolites in plasma, bile, urine and feces of normal and blood stasis rats after oral administration of hydroxysafflor yellow A. J. Chromatogr. B.

[39] Xu R.-a., Xu Z.-s., Ge R.-s. (2014). Effects of hydroxysafflor yellow A on the activity and mRNA expression of four CYP isozymes in rats. J. Ethnopharmacol..

[40] Wu S., Gao J. (2026). Diabetic Retinopathy: Research Advances in Canonical Cellular Pathways and Emerging Mechanisms. Exp. Eye Res..

[41] Mimura T., Noma H. (2025). Oxidative stress in diabetic retinopathy: A comprehensive review of mechanisms, biomarkers, and therapeutic perspectives. Antioxidants.

[42] He W., Tang P., Lv H. (2025). Targeting oxidative stress in diabetic retinopathy: Mechanisms, pathology, and novel treatment approaches. Front. Immunol..

[43] Itoh K., Wakabayashi N., Katoh Y., Ishii T., Igarashi K., Engel J.D., Yamamoto M. (1999). Keap1 represses nuclear activation of antioxidant responsive elements by Nrf2 through binding to the amino-terminal Neh2 domain. Genes Dev..

[44] Yang Y., Zou H. (2025). Research progress on Nrf2 intervention in the treatment of diabetic retinopathy. Front. Endocrinol..

[45] Kinuthia U.M., Wolf A., Langmann T. (2020). Microglia and inflammatory responses in diabetic retinopathy. Front. Immunol..

[46] Rübsam A., Parikh S., Fort P.E. (2018). Role of inflammation in diabetic retinopathy. Int. J. Mol. Sci..

[47] Jiao X., Tian G. (2025). Role of the NLRP3 inflammasome in diabetes and its complications. Mol. Med. Rep..

[48] Yao M., Liu Y., Meng D., Zhou X., Chang D., Li L., Wang N., Huang Q. (2025). Hydroxysafflor yellow A attenuates the inflammatory response in cerebral ischemia–reperfusion injured mice by regulating microglia polarization per SIRT1-mediated HMGB1/NF-κB signaling pathway. Int. Immunopharmacol..

[49] Maugeri G., Bucolo C., Drago F., Rossi S., Di Rosa M., Imbesi R., D’Agata V., Giunta S. (2021). Attenuation of high glucose-induced damage in RPE cells through p38 MAPK signaling pathway inhibition. Front. Pharmacol..

[50] Du Y., Tang J., Li G., Berti-Mattera L., Lee C.A., Bartkowski D., Gale D., Monahan J., Niesman M.R., Alton G. (2010). Effects of p38 MAPK inhibition on early stages of diabetic retinopathy and sensory nerve function. Investig. Ophthalmol. Vis. Sci..

[51] Wu J., Gorman A., Zhou X., Sandra C., Chen E. (2002). Involvement of caspase-3 in photoreceptor cell apoptosis induced by in vivo blue light exposure. Investig. Ophthalmol. Vis. Sci..

[52] Perche O., Doly M., Ranchon-Cole I. (2007). Caspase-dependent apoptosis in light-induced retinal degeneration. Investig. Ophthalmol. Vis. Sci..

[53] Huang P., Wu S.-p., Wang N., Seto S., Chang D. (2021). Hydroxysafflor yellow A alleviates cerebral ischemia reperfusion injury by suppressing apoptosis via mitochondrial permeability transition pore. Phytomedicine.

[54] Yu L., Jin Z., Li M., Liu H., Tao J., Xu C., Wang L., Zhang Q. (2022). Protective potential of hydroxysafflor yellow A in cerebral ischemia and reperfusion injury: An overview of evidence from experimental studies. Front. Pharmacol..

[55] Ruan J., Wang L., Wang N., Huang P., Chang D., Zhou X., Seto S., Li D., Hou J. (2025). Hydroxysafflor Yellow A promotes angiogenesis of brain microvascular endothelial cells from ischemia/reperfusion injury via glycolysis pathway in vitro. J. Stroke Cerebrovasc. Dis..

[56] Lai Z., Li C., Ma H., Hua S., Liu Z., Huang S., Liu K., Li J., Feng Z., Cai Y. (2023). Hydroxysafflor yellow a confers neuroprotection against acute traumatic brain injury by modulating neuronal autophagy to inhibit NLRP3 inflammasomes. J. Ethnopharmacol..

[57] Ye J., Lu S., Wang M., Ge W., Liu H., Qi Y., Fu J., Zhang Q., Zhang B., Sun G. (2020). Hydroxysafflor Yellow A Protects Against Myocardial Ischemia/Reperfusion Injury via Suppressing NLRP3 Inflammasome and Activating Autophagy. Front. Pharmacol..

[58] Chen G., Li C., Zhang L., Yang J., Meng H., Wan H., He Y. (2022). Hydroxysafflor yellow A and anhydrosafflor yellow B alleviate ferroptosis and parthanatos in PC12 cells injured by OGD/R. Free Radic. Biol. Med..

[59] Venkatesan A., Bernstein A.M. (2025). Protein misfolding and mitochondrial dysfunction in glaucoma. Front. Cell Dev. Biol..

[60] Harun-Or-Rashid M., Zhang J., Frikke-schmidt H., Wong W. (2024). Overexpression of hGDF15 in retina of transgenic mice is associated with retinal ganglion cell neuroprotection in a murine glaucoma model of increased intraocular pressure. Investig. Ophthalmol. Vis. Sci..

[61] Mimura T., Noma H. (2025). Title Oxidative Stress in Age-Related Macular Degeneration: From Molecular Mechanisms to Emerging Therapeutic Targets. Antioxidants.

[62] Adijanto J., Du J., Moffat C., Seifert E.L., Hurley J.B., Philp N.J. (2014). The retinal pigment epithelium utilizes fatty acids for ketogenesis. J. Biol. Chem..

[63] Sparrow J.R., Nakanishi K., Parish C.A. (2000). The lipofuscin fluorophore A2E mediates blue light-induced damage to retinal pigmented epithelial cells. Investig. Ophthalmol. Vis. Sci..

[64] Kurihara T., Westenskow P.D., Gantner M.L., Usui Y., Schultz A., Bravo S., Aguilar E., Wittgrove C., Friedlander M.S.H., Paris L.P. (2016). Hypoxia-induced metabolic stress in retinal pigment epithelial cells is sufficient to induce photoreceptor degeneration. eLife.

[65] Kato Y., Oguchi Y., Omori T., Kasai A., Ogasawara M., Sugano Y., Itagaki K., Ojima A., Ishida Y., Machida T. (2022). Age-Related Maculopathy Susceptibility 2 and Complement Factor H Polymorphism and Intraocular Complement Activation in Neovascular Age-Related Macular Degeneration. Ophthalmol. Sci..

[66] Kalesnykas G., Oglesby E.N., Zack D.J., Cone F.E., Steinhart M.R., Tian J., Pease M.E., Quigley H.A. (2012). Retinal ganglion cell morphology after optic nerve crush and experimental glaucoma. Investig. Ophthalmol. Vis. Sci..

[67] Sappington R.M., Carlson B.J., Crish S.D., Calkins D.J. (2010). The microbead occlusion model: A paradigm for induced ocular hypertension in rats and mice. Investig. Ophthalmol. Vis. Sci..

[68] Williams P.A., Harder J.M., Foxworth N.E., Cochran K.E., Philip V.M., Porciatti V., Smithies O., John S.W.M. (2017). Vitamin B 3 modulates mitochondrial vulnerability and prevents glaucoma in aged mice. Science.

[69] Qiu F., Meng T., Chen Q., Zhou K., Shao Y., Matlock G., Ma X., Wu W., Du Y., Wang X. (2019). Fenofibrate-Loaded Biodegradable Nanoparticles for the Treatment of Experimental Diabetic Retinopathy and Neovascular Age-Related Macular Degeneration. Mol. Pharm..

[70] Park D.Y., Lee J., Kim J., Kim K., Hong S., Han S., Kubota Y., Augustin H.G., Ding L., Kim J.W. (2017). Plastic roles of pericytes in the blood-retinal barrier. Nat. Commun..

[71] Varela-Fernández R., Díaz-Tomé V., Luaces-Rodríguez A., Conde-Penedo A., García-Otero X., Luzardo-Álvarez A., Fernández-Ferreiro A., Otero-Espinar F.J. (2020). Drug delivery to the posterior segment of the eye: Biopharmaceutic and pharmacokinetic considerations. Pharmaceutics.

[72] Tang D., Ye T., Chen X., Yang J., Xie Y. (2023). Transepithelial transport characteristics of Hydroxysafflor yellow A across cellular monolayers and the effects of the influx and efflux transporters. Food Biosci..

[73] Li Y., Zhang Z.y., Zhang J.l. (2007). Determination of hydroxysafflor yellow A in rat plasma and tissues by high-performance liquid chromatography after oral administration of safflower extract or safflor yellow. Biomed. Chromatogr..

[74] Gao H., Zhou H.-M., Yue S.-J., Feng L.-M., Guo D.-Y., Li J.-J., Zhao Q., Huang L., Tang Y.-P. (2022). Oral bioavailability-enhancing and anti-obesity effects of hydroxysafflor yellow A in natural deep eutectic solvent. ACS Omega.

[75] Zhang Y., Zhong C., Wang Q., Zhang J., Zhao H., Huang Y., Zhao D., Yang J. (2023). Nanoemulsions of hydroxysafflor yellow a for enhancing physicochemical and in vivo performance. Int. J. Mol. Sci..

[76] Chen Y., Li M., Wen J., Pan X., Deng Z., Chen J., Chen G., Yu L., Tang Y., Li G. (2022). Pharmacological Activities of Safflower Yellow and Its Clinical Applications. Evid.-Based Complement. Altern. Med..

[77] Chrai S.S., Patton T.F., Mehta A., Robinson J.R. (1973). Lacrimal and Instilled Fluid Dynamics in Rabbit Eyes. J. Pharm. Sci..

[78] Ahmed I., Patton T.F. (1985). Importance of the noncorneal absorption route in topical ophthalmic drug delivery. Investig. Ophthalmol. Vis. Sci..

[79] Djebli N., Khier S., Griguer F., Coutant A.-L., Tavernier A., Fabre G., Leriche C., Fabre D. (2016). Ocular Drug Distribution After Topical Administration: Population Pharmacokinetic Model in Rabbits. Eur. J. Drug Metab. Pharmacokinet..

[80] Nian S., Lo A.C.Y., Mi Y., Ren K., Yang D. (2021). Neurovascular unit in diabetic retinopathy: Pathophysiological roles and potential therapeutical targets. Eye Vis..

[81] Ren J., Zhang S., Pan Y., Jin M., Li J., Luo Y., Sun X., Li G. (2022). Diabetic retinopathy: Involved cells, biomarkers, and treatments. Front. Pharmacol..

[82] Xia T., Rizzolo L.J. (2017). Effects of diabetic retinopathy on the barrier functions of the retinal pigment epithelium. Vis. Res..

[83] Li Y., Faiz A., Moshage H., Schubert R., Schilling L., Kamps J.A. (2021). Comparative transcriptome analysis of inner blood-retinal barrier and blood–brain barrier in rats. Sci. Rep..

[84] Chapy H., Saubaméa B., Tournier N., Bourasset F., Behar-Cohen F., Declèves X., Scherrmann J.M., Cisternino S. (2016). Blood-brain and retinal barriers show dissimilar ABC transporter impacts and concealed effect of P-glycoprotein on a novel verapamil influx carrier. Br. J. Pharmacol..

[85] Nomoto H., Shiraga F., Kuno N., Kimura E., Fujii S., Shinomiya K., Nugent A.K., Hirooka K., Baba T. (2009). Pharmacokinetics of bevacizumab after topical, subconjunctival, and intravitreal administration in rabbits. Investig. Ophthalmol. Vis. Sci..

[86] Wang Y., Hu Y., An J., Zhang H., Liu X., Li X., Zhang Z., Zhang X. (2024). Liposome-Based Permeable Eyedrops for Effective Posterior Segment Drug Delivery. Adv. Funct. Mater..

[87] Muya L., Kansara V., Cavet M.E., Ciulla T. (2022). Suprachoroidal Injection of Triamcinolone Acetonide Suspension: Ocular Pharmacokinetics and Distribution in Rabbits Demonstrates High and Durable Levels in the Chorioretina. J. Ocul. Pharmacol. Ther..

[88] Reitan G., Kjellevold Haugen I.B., Andersen K., Bragadottir R., Bindesbøll C. (2023). Through the Eyes of Patients: Understanding Treatment Burden of Intravitreal Anti-VEGF Injections for nAMD Patients in Norway. Clin. Ophthalmol..

[89] Lee S., Park J.Y., Hong H.K., Son J.Y., Kim B., Chung J.Y., Woo S.J., Park K.D. (2024). Intravitreal long-term sustained ranibizumab delivery using injectable microgel-embedded hydrogel. Asian J. Pharm. Sci..

[90] Hu L., Xu Y., Meng H. (2022). Development and Evaluation of Puerarin Loaded-Albumin Nanoparticles Thermoresponsive in situ Gel for Ophthalmic Delivery. Drug Des. Dev. Ther..

[91] Dahiya P., Zafar A., Ahmad F.J., Khalid M., Ali A. (2023). Development of Forskolin and rutin-loaded polymeric nanoparticles for enhancement of topical ocular delivery: Optimization, in-vitro, ex-vivo, and toxicity evaluation. J. Drug Deliv. Sci. Technol..

[92] Gözcü S., Polat H.K., Gültekin Y., Ünal S., Karakuyu N.F., Şafak E.K., Doğan O., Pezik E., Haydar M.K., Aytekin E. (2024). Formulation of hesperidin-loaded in situ gel for ocular drug delivery: A comprehensive study. J. Sci. Food Agric..

[93] Siafaka P., Yağcılar A.P.Y., Güven G.K., Yoltaş A., Okur N.Ü. (2024). In situ gels loaded with naringin as ocular drug delivery carriers; development and preliminary characterization. J. Res. Pharm..

[94] Zhu L., Li N., Zhang R., Shao Y., Lai J. (2026). Therapeutic Effect of Baicalin-Loaded Thermosensitive Liposomal Hydrogel in Autoimmune Uveitis. ACS Omega.

[95] U.S. Food and Drug Administration (2018). Liposome Drug Products: Chemistry, Manufacturing, and Controls; Human Pharmacokinetics and Bioavailability; and Labeling Documentation; Guidance for Industry; Availability. Fed. Regist..

[96] Renukuntla J., Palakurthi S.S., Bolla P.K., Clark B.A., Boddu S.H.S., Manda P., Sockwell S., Charbe N.B., Palakurthi S. (2022). Advances in in-vitro bioequivalence testing methods for complex ophthalmic generic products. Int. J. Pharm..

[97] Roy Chowdhury U., Pervan-Steel C.L., Sheeler R., Sookdeo H.K., Rogers B., Casale R., Dosa P.I., Htoo T., Wirostko B.M., Fautsch M.P. (2023). Preclinical Pharmacokinetic Profile of Topical Ophthalmic and Intravenous Delivery of QLS-101, a Novel ATP-Sensitive Potassium Channel Opening Ocular Hypotensive Agent. J. Ocul. Pharmacol. Ther..

[98] Valtari A., Posio S., Toropainen E., Balla A., Puranen J., Sadeghi A., Ruponen M., Ranta V.-P., Vellonen K.-S., Urtti A. (2024). Comprehensive ocular and systemic pharmacokinetics of dexamethasone after subconjunctival and intravenous injections in rabbits. Eur. J. Pharm. Biopharm..

[99] Rimpelä A.-K., Reunanen S., Hagström M., Kidron H., Urtti A. (2018). Binding of Small Molecule Drugs to Porcine Vitreous Humor. Mol. Pharm..

[100] Grove K.J., Kansara V., Prentiss M., Long D., Mogi M., Kim S., Rudewicz P.J. (2017). Application of Imaging Mass Spectrometry to Assess Ocular Drug Transit. SLAS Discov. Adv. Sci. Drug Discov..

[101] Hu Z.-C., Xie Z.-J., Tang Q., Li X.-B., Fu X., Feng Z.-H., Xuan J.-W., Ni W.-F., Wu A.-M. (2018). Hydroxysafflor yellow A (HSYA) targets the NF-κB and MAPK pathways and ameliorates the development of osteoarthritis. Food Funct..

[102] Xu X., Guo Y., Zhao J., Wang N., Ding J., Liu Q. (2016). Hydroxysafflor Yellow A Inhibits LPS-Induced NLRP3 Inflammasome Activation via Binding to Xanthine Oxidase in Mouse RAW264.7 Macrophages. Mediat. Inflamm..

[103] Ge C., Peng Y., Li J., Wang L., Zhu X., Wang N., Yang D., Zhou X., Chang D. (2023). Hydroxysafflor Yellow a Alleviates Acute Myocardial Ischemia/Reperfusion Injury in Mice by Inhibiting Ferroptosis via the Activation of the HIF-1α/SLC7A11/GPX4 Signaling Pathway. Nutrients.

[104] Yang Y., Lin Y., Han Z., Wang B., Zheng W., Wei L. (2024). Ferroptosis: A novel mechanism of cell death in ophthalmic conditions. Front. Immunol..

[105] Wang H.-F., Ma J.-X., Shang Q.-L., An J.-B., Chen H.-T., Wang C.-X. (2019). Safety, pharmacokinetics, and prevention effect of intraocular crocetin in proliferative vitreoretinopathy. Biomed. Pharmacother..

